# Reliability of the AI-Assisted Assessment of the Proximity of the Root Apices to Mandibular Canal

**DOI:** 10.3390/jcm13123605

**Published:** 2024-06-20

**Authors:** Wojciech Kazimierczak, Natalia Kazimierczak, Kamila Kędziora, Marta Szcześniak, Zbigniew Serafin

**Affiliations:** 1 Department of Radiology and Diagnostic Imaging, Collegium Medicum, Nicolaus Copernicus University in Torun, Jagiellońska 13-15, 85-067 Bydgoszcz, Poland; 2 Kazimierczak Private Medical Practice, Dworcowa 13/u6a, 85-009 Bydgoszcz, Poland; kamila.kedziora@cm.umk.pl (K.K.); serafin@cm.umk.pl (Z.S.); 3 Chair of Practical Clinical Dentistry, Department of Diagnostics, Poznań University of Medical Sciences, Fredry 10, 61-701 Poznań, Poland; mszczesniak1993@gmail.com

**Keywords:** artificial intelligence (AI), dental imaging, computed tomography, diagnostic test accuracy, automatic detection, mandibular canal, root apex

## Abstract

**Background**: This study evaluates the diagnostic accuracy of an AI-assisted tool in assessing the proximity of the mandibular canal (MC) to the root apices (RAs) of mandibular teeth using computed tomography (CT). **Methods**: This study involved 57 patients aged 18–30 whose CT scans were analyzed by both AI and human experts. The primary aim was to measure the closest distance between the MC and RAs and to assess the AI tool’s diagnostic performance. The results indicated significant variability in RA-MC distances, with third molars showing the smallest mean distances and first molars the greatest. Diagnostic accuracy metrics for the AI tool were assessed at three thresholds (0 mm, 0.5 mm, and 1 mm). **Results**: The AI demonstrated high specificity but generally low diagnostic accuracy, with the highest metrics at the 0.5 mm threshold with 40.91% sensitivity and 97.06% specificity. **Conclusions**: This study underscores the limited potential of tested AI programs in reducing iatrogenic damage to the inferior alveolar nerve (IAN) during dental procedures. Significant differences in RA-MC distances between evaluated teeth were found.

## 1. Introduction

The mandible is the only movable bone of the facial skeleton, facilitating the functions of speech, mastication, and facial expression. The mandibular canal (MC) is a bony canal located bilaterally in the body and ramus of the mandible, beginning at the mandibular foramen in the ramus area and ending at the mental foramen in the second premolar area. The MC contains the inferior alveolar artery (IAA), inferior alveolar vein, and inferior alveolar nerve (IAN). The IAA and IAN provide branches that innervate and supply blood to the dental pulp of teeth in this area [1,2]. The IAN is part of the mandibular branch of the trigeminal nerve and provides sensory innervation to the teeth, chin, and lower lip.

The relationships between the localization of the MC and the RAs of mandibular teeth are particularly important due to the risk of damage to the IAN during dental procedures. The IAN is the most injured nerve (64.4%) among all branches of the trigeminal nerve [3]. Most of the injuries are iatrogenic and are attributed to various dental interventions, including implantology, surgical, orthodontic, and endodontic procedures [4,5,6,7,8]. IAN damage can occur during dental procedures, usually through two mechanisms: direct mechanical damage or indirect damage through factors such as the accumulation of a hematoma, chemical or thermal irritation, or inflammation [4]. The reported rate of IAN injury after third molar extractions is up to 8% [9], and IAN injury accounts for the vast majority of IAN damage [5]. Altered sensation has been reported in 13% of patients after mandibular implant surgery [10]. Endodontic treatment accounts for 8 to 35% of reported IAN iatrogenic damage [5,11], with an incidence of up to 10% at the individual patient level [12].

Damage to the IAN can result in paresthesia of the lower lip region, anesthesia, and trigeminal neuralgia, diminishing quality of life [6,13]. Pain typically affects the area innervated by the IAN, as well as radiates to the ear and neck regions [14]. Disturbed nerve conduction may also manifest as loss of sensation (anesthesia), decreased sensation (hypoesthesia), increased perception of stimuli (hyperesthesia), and sensation of stimuli without a physical cause (paresthesia) [14]. Patients may suffer from varying degrees of symptoms, including numbness in the lower teeth, chin, and lower lip, leading to biting injuries, problems with chewing, an inability to control food and liquid with unnoticed drooling, difficulties with speech, and occasional chronic painful conditions such as allodynia [11]. Although most complications are mild and transient, up to 19.6% of neurosensory disturbances are permanent [11].

The risk of inferior alveolar nerve (IAN) damage during dental procedures is influenced by several factors, including the practitioner’s experience, the patient’s age, and the anatomical position and course of the MC [15,16]. Precise determination of the canal pathway is possible through imaging studies, with particular emphasis on three-dimensional (3D) techniques such as computed tomography (CT) and cone-beam computed tomography (CBCT). These modalities enable the precise assessment of MC structure, including anatomical variants [17,18]. The significance of meticulous treatment planning, which includes the use of radiographs to assess bone volume, morphology, and neurovascular structures, is crucial for preventing nerve injuries [19]. Digital dentistry has accelerated the processes of diagnosis and treatment planning, becoming an essential component of advanced, contemporary dental care today.

Recent advancements in AI technology in orthodontics and dental radiology have led to the creation of several AI-powered programs, such as CephX (ORCA Dental AI, Las Vegas, NV, USA), which facilitates automated cephalometric analyses (CAs). CephX’s website briefly informs the users that the program uses innovative AlgoCeph^®^ technology and that the program is HIPAA-compliant and FDA-approved. To date, studies have reported the relatively high accuracy and reliability of AI platforms in maxillofacial radiology [20,21]. However, some studies have highlighted the programs’ low reliability in assessing skeletal asymmetry [22]. One significant module of CephX is the automated detection of the root apex (RA)-MC proximity in CT/CBCT images. This module automatically alerts the user to the proximity of these two structures. In practice, such a module could benefit both clinicians and patients, potentially leading to modifications in surgical or endodontic techniques to reduce the risk of iatrogenic damage to the IAN.

The first aim of the present study was to assess the closest distance between the MC and the RAs of mandibular second premolars and molars using CT. The second aim was to assess the diagnostic accuracy of the tested AI platform for detecting the proximity of the RAs and MC.

## 2. Materials and Methods

### 2.1. Patients, Sample Size Calculation

The study population consisted of 60 consecutive patients (40 males and 20 females, aged 18–30) admitted to the Emergency Department of University Hospital No. 1 in Bydgoszcz, Poland, between 1 January 2020 and 31 December 2022. CT scans were acquired in a range covering the entire craniofacial area.

The indications for CT scans included post-traumatic assessments in patients who experienced generalized trauma or trauma to the craniofacial area.

The inclusion criteria were as follows:

1. Aged 18–30 years to minimize the risk of the presence of dental prostheses, implants, and multiple missing teeth.
2. Centric occlusion of the patient’s teeth.
3. CT scan covering the region from the chin to the vertex.

The exclusion criteria were as follows:

1. Severe motion and metal artifacts.
2. Fractures of the mandible.
3. Four teeth missing per dental arch.
4. Tumors in the craniofacial area.
5. Overall poor image quality.

The sample size was calculated to achieve a power of 80% (β = 0.20) at a 5% significance level (α = 0.05) to detect a difference in the proportion of successful outcomes between groups. https://clincalc.com/ (accessed on 4 May 2024). An online calculator was used to define the sample size.

### 2.2. Image Acquisition and Postprocessing

All CT images were acquired with a 64-slice CT scanner (Discovery 750HD; GE Health Care; Waukesha, WI, USA). The following parameters were utilized: 64 × 0.625 mm collimation, 32 cm scan FOV, 260 mA tube current, 120 kVp tube voltage, 0.625 mm slice thickness, 0.8 s per gantry rotation, and 0.531 pitch. All the CT images were reconstructed with a slice thickness of 0.6 mm. Patient identifiers were removed to maintain anonymity, and images were coded for blinded analysis.

### 2.3. AI Evaluation

The images were uploaded to the cloud-based commercially available platform CephX. After the initial analysis, the AI software automatically provided warnings for the RA-MC proximity.

### 2.4. Multireader Evaluation

**Figure 1:**
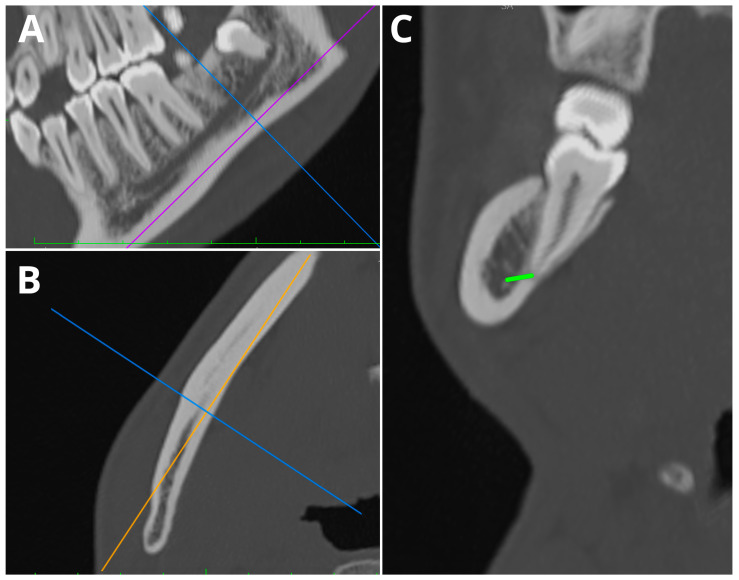
Sample case of measurements of RA-MC distance of tooth 47 using MPRs. (**A**) Sagittal plane; (**B**) axial plane; (**C**) coronal plane; RA-MC distance marked with green color.

The images were independently evaluated by two readers—a radiographer and a maxillofacial surgeon. Both readers independently assessed CT images, and both were blinded to the second reader’s results and the results of the AI-automated analysis. The minimal distances between the RAs and the second premolars, molars, and the MC were evaluated within the whole study group by both readers. During the measurements, the images were evaluated using multi-planar reconstructions (MPRs). The image was aligned to the long axis of MC, and the images were evaluated frontal, perpendicular to the long axis of the MC plane. The MC was traced from the mental foramen to the distal roots of the third molars. The shortest distance between the most proximal to the MC–RA and the border of the MC was measured. In the absence of the tooth, no measurements were taken. In the case of direct communication between the RAs and the MC, a value of 0 was assigned. The average RA-MC distances were calculated. Figure 1 presents the method of measurement undertaken by both of the readers. 

After completing the measurements, the senior reader (a radiologist with 8 years of experience in CT assessment) evaluated the results and verified the accuracy of the findings, which indicated a reduced RA-MC distance (<1 mm).

### 2.5. The Inter- and Intrareader Concordance of the Results

To maintain consistency in evaluations, the senior reader instructed both readers on how to execute RA-MC measurements. The concordance between the readers’ measurements was calculated to assess the interreader concordance of the results. 

CT images of 19 patients in the final study group (33%) were manually uploaded to the CephX database as new patients to assess the repeatability of the program’s assessments.

### 2.6. Statistical Evaluation

The diagnostic accuracy of the AI for the reduced RA-MC was assessed by comparison to the reference standard set by a senior reader. The sensitivity, specificity, accuracy, positive predictive value (PPV), negative predictive value (NPV), and F1 score were calculated. The interrater reliability of measurements between two readers was assessed with the Fleiss kappa. The significance level was set to 0.05. All the analyses were conducted in R software, version 4.3.2.

## 3. Results

### 3.1. Population, Sample Size Calculations

Three patients were excluded from the initial study group because they failed to meet our eligibility criteria (all three with at least four teeth missing in the mandible). The authors reviewed the CT scans of 57 patients. The mean age of all participants was 22.7 years (range 18–30). A total of 18 females with a mean age of 22.5 years and 39 males with a mean age of 22.9 years were included.

The sample size was determined restrospectively with following diagnostic accuracy metrics in RA-MC proximity detection: sensitivity of AI and readers 40% and 90% respectively. Assuming these parameters, the minimum sample size calculated was 11. The sample size calculations ensured that the study had sufficient power to detect meaningful differences in diagnostic performance.

### 3.2. Mean RA-MC Distances

**Figure 2:**
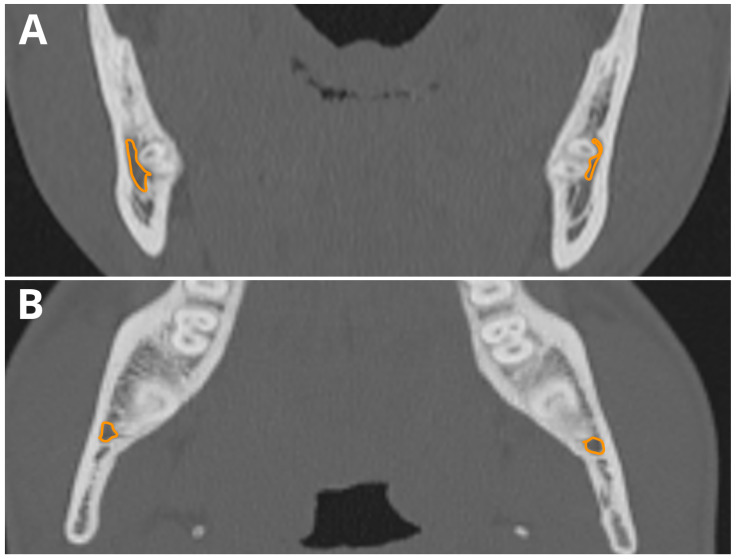
Bilateral direct RA-MC communication of third molars. (**A**) Axial plane; (**B**) coronal plane. The course of MC is marked in orange. RA-MC proximity detected by CephX.

Table 1 and Table 2 summarize the results of both readers’ assessments. The smallest mean distances between teeth and the MC were found for the third molars, and the greatest distances were found for the first molars. Moreover, third molars were the most frequently missing (extracted) teeth—up to 12 in the case of 48 teeth. The frequency of direct communication between the RAs and MC was highest in the case of third molars, up to 33% in the case of tooth 48. A sample case with bilateral RA-MC communication is shown in Figure 2. 

### 3.3. Diagnostic Accuracy of AI

**Figure 3:**
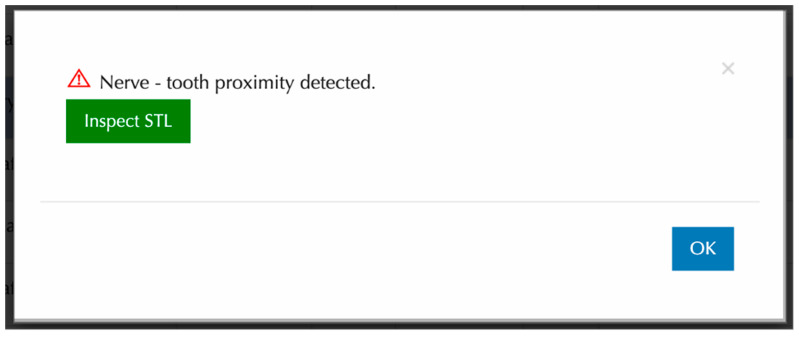
Sample RA-MC proximity alert provided by CephX.

**Figure 4:**
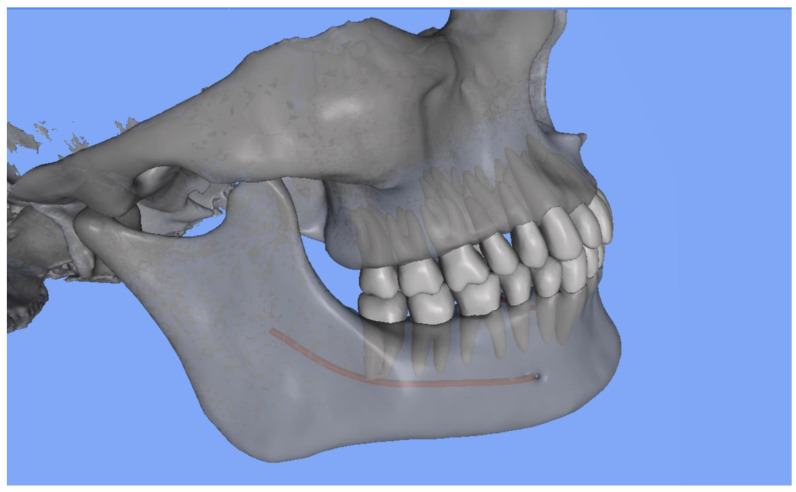
Three-dimensional model presenting teeth and MC segmentation results. RA-MC proximity detected by AI program.

CephX reports on RA-MC proximity contained only warnings about the potential proximity of these structures. They did not include any data indicating the distances between the root apices concerned by the potential RA-MC proximity, nor did they specify the side on which the diagnosed RA-MC proximity was present. However, the program provided the automatically generated STL, enabling visual inspection of segmented teeth and the MC. Figure 3 presents the information provided by the AI program. Figure 4 presents a three-dimensional model of craniofacial bones and teeth generated by CephX.

**Figure 5:**
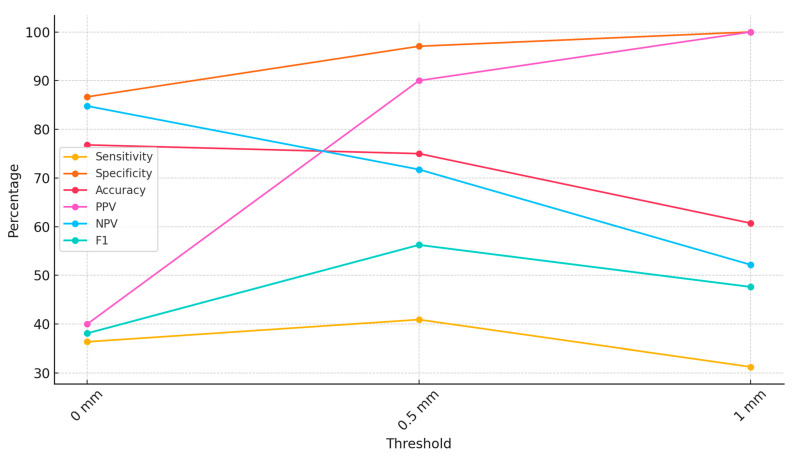
Diagnostic accuracy metrics of AI program for RA-MC proximity.

Since the AI program did not state the criterion for RA-MC proximity alerts, three thresholds were analyzed (0 mm, 0.5 mm, and 1.0 mm) to assess the accuracy of CephX diagnoses of RA-MC proximity. In the case of RA-MC distances of any analyzed patient’s tooth reported by the readers (confirmed by a senior reader) below 1 mm, 0.5 mm, and/or equal to 0, the diagnosis of RA-MC proximity was considered positive. The detailed results of the program’s diagnostic accuracy are presented in Table 3 and in Figure 5.

**Figure 6:**
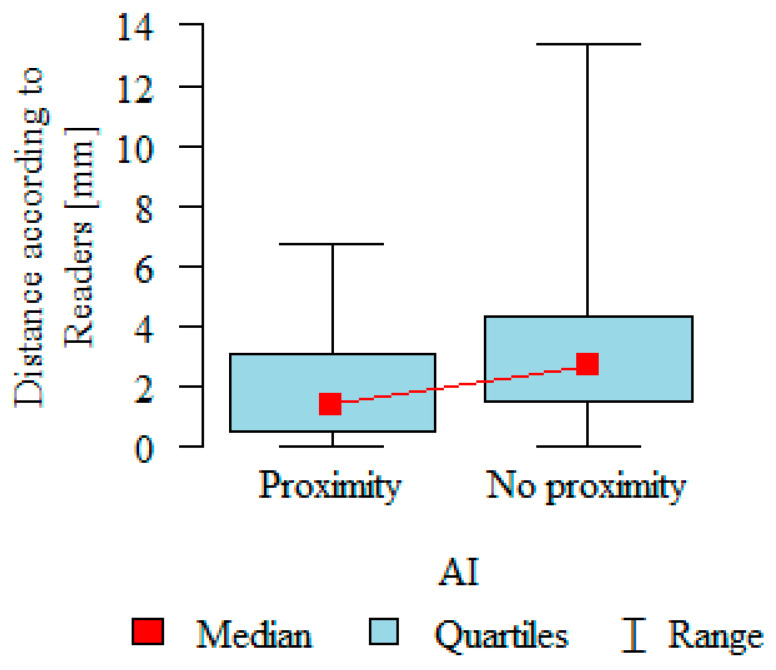
Correlation between mean RA-MC distances and AI’s diagnosis on RA-MC proximity.

The RA-MC distances in the readers’ evaluation were significantly greater when AI did not indicate a reduced distance compared to when AI indicated a reduced distance (Table 4 and Figure 6).

### 3.4. The Inter- and Intrareader Agreement of the Results

The results of interreader concordance on assessments of RA-MC distances performed by the human readers showed excellent agreement (Table 5).

The analysis of the repeatability of AI’s diagnoses on RA-MC proximity showed differences in part of the repeated analyses. However, the results of the repeatability of the AI assessments of the RA-MC proximity showed strong agreement (Table 6).

## 4. Discussion

This study aimed to evaluate the closest distance between the RAs and the MC using CT scans and to assess the diagnostic accuracy of the AI platform in detecting RA-MC proximity. This study found significant differences in RA-MC distances among mandibular teeth. The findings of this study demonstrate the moderate diagnostic accuracy of the AI-assisted CephX platform in assessing the proximity of the MC to the RA of mandibular teeth. The results underscore that the evaluated AI platform exhibits limited potential in clinical settings.

The relationship between the RAs of mandibular teeth and the MC is crucial for dental procedures to avoid IAN damage. The results of our study showed the lowest mean distances between the RAs of the third molars and the MC and the greatest distances between the first molars and the MC. A systematic review by Puciło et al. [23] analyzed the mean distances between the RAs of mandibular teeth and the MC. The authors showed slightly different results, with the shortest distances to the second premolars, first molars, second molars, and third molars being 1.65 mm, 1.23 mm, 0.64 mm, and 1.28 mm, respectively. However, there are also studies confirming our results with the greatest distances between RAs of first molars and the MC [24,25]. As studies conducted on large cohorts have shown, the RA-MC distances were generally shorter in females and younger individuals, with significant differences noted in patients younger than 35 years compared to those in older populations [23,25,26,27]. Kawashima et al. concluded that these phenomena suggest the possibility of increased bone growth after tooth eruption and/or MC inferior migration with age [28]. 

The sensitivity and specificity of the AI platform varied depending on the applied distance threshold. For a threshold of 0.5 mm, the AI demonstrated a sensitivity of 40.91% and a specificity of 97.06%, with an accuracy of 75%. Our findings show that while the AI platform can be highly specific in detecting close proximities, it may be overly conservative in terms of diagnosis, leading to low sensitivity. Therefore, F1 scores remain low despite high specificity. There is suspicion that the results of our study may be due to the use of CT images instead of CBCT images; however, to the best of the authors’ knowledge, no studies to date have been published analyzing the diagnostic accuracy of CephX in CBCT image assessment. Image quality alterations such as noise, object contrast, and artifacts are known factors hampering the segmentation process [29,30]. Since, in general, CT images exhibit lower resolution than contemporary CBCT scan measurements [31,32,33], the CT origin of analyzed datasets may influence the results of our analysis. Several additional factors have likely contributed to CephX’s low sensitivity in assessing RA-MC proximity, with the most significant being probable algorithm limitations. The AI algorithm used by CephX may have inherent limitations in accurately detecting the proximity of complex anatomical structures like the RA and MC. The existence of these limitations is indicated by the significant variability in diagnoses. The study found variability in repeated analyses of RA-MC proximity by CephX, suggesting inconsistencies in the AI’s performance. This variability can lead to a lower overall sensitivity, as the AI might miss detections in some instances due to inconsistent performance. Previous studies on CephX’s performance in other applications, such as cephalometric analysis, have shown that manual corrections are often necessary to achieve clinically acceptable results [34]. This reliance on manual adjustments indicates that the AI might not be fully reliable on its own, contributing to its low sensitivity in detecting RA-MC proximity.

These results are comparable to findings from previous studies on CephX applications in CT dental imaging, which have shown similar ranges of diagnostic performance in various applications [22,35]. Two 2023 studies have evaluated CephX performance in different CT image assessments: skeletal facial asymmetry assessment and the repeatability of automated cephalometric analysis. The authors showed that despite the reproducibility of the multiparametric cephalometric analysis being excellent for most of the parameters, three angular measurements exhibited poor reproducibility [35]. Another study on the CephX application in facial asymmetry assessment showed no agreement between the results of manual and automated AI analyses and a significant number of evidently erroneous cephalometric tracings [22].

Besides the above-mentioned studies, to the best of the authors’ knowledge, no studies were conducted on CephX utilization in CT or CBCT examinations. However, there are some studies published analyzing the main application of CephX—automated cephalometric analysis on lateral cephalograms. All the studies showed some inaccuracies in the results of automated cephalometric analyses conducted with CephX. Despite these issues, the authors found the program useful [20,35,36]. The study by Meric and Naoumova [20] investigated the application of AI analysis based on lateral cephalograms of 40 patients preceding orthodontic treatment. The authors compared the results of eight angular and four linear parameters, measured by a single researcher using three methods: manually with Dolphin Imaging 13.01 software and automatically with CephNinja 3.51 and CephX. The cephalometric landmarks defined by CephX were manually corrected. The study showed that CephX had the highest variability in results, with significant deviations in several cephalometric measurements. After landmark correction, CephX’s results did not significantly differ from those of the other two programs. A notable advantage of CephX was the significant reduction in analysis time despite the need for manual correction. A study by Khalid and Azeez [36] analyzed results of cephalometric analysis based on 14 measurements on 25 lateral cephalograms, comparing an expert’s measurements with CephX results. A high level of agreement was demonstrated, except for measurements of two measurements. It was concluded that the results of the program’s analyses are suitable for clinical purposes and comparable to expert results. The study by Alqahtani [35] assessed the repeatability of cephalometric measurements using CephX and FACAD. Thirty radiograms were analyzed, focusing on 16 cephalometric landmarks and 16 linear and angular measurements. Statistical analysis of the results showed no significant differences between the programs, except for three measurements. The study’s author stated that both platforms achieve highly consistent results, with clinically insignificant differences. 

Since the CephX developer does not inform users regarding the method of RA-MC proximity detection and the defined criteria for positive diagnosis, we have examined the program’s diagnostic parameters using three thresholds. The analysis of diagnostic accuracy revealed that the AI platform performed best at a 0.5 mm threshold in terms of balancing sensitivity and specificity. At 1 mm, the specificity was maximized, but the sensitivity decreased, while at 0 mm, the sensitivity was highest, but the specificity decreased. The adopted thresholds, in our opinion, exhibit variations in the diagnostic accuracy of the tested AI platform. As shown in Figure 3, the dependence of accuracy metrics on the predefined threshold clearly indicates that further application of larger thresholds (1.5, 2 mm, etc.) would lead to a further decrease in sensitivity and F1 score.

The primary clinical implication of this study is the potential reduction in the risk of iatrogenic damage to the IAN during dental procedures. Previous studies have reported varying rates of IAN damage, with third molar extractions posing a significant risk [7,10]. Moreover, dental implantology is an area where the localization of the MC must be meticulously examined before the procedure. An implant MC distance of 1–1.5 mm is suggested to prevent IAN damage caused by dental implants [37]. Detailed knowledge of the MCl’s location relative to the tooth roots aids in planning the surgical access line and avoiding complications during endodontic surgery [38]. Overfilling of endodontic materials into the MC can lead to nerve damage, resulting in persistent anesthesia or paresthesia. This highlights the importance of precise assessment and careful execution of endodontic procedures [39]. However, manual labeling of the course of the IAN is very labor-intensive and time-consuming [40]. Additionally, there is significant anatomical variability in the root canal systems of mandibular teeth, which can affect the proximity to the MC [41]. Direct communication between RAs and the mandibular canal is common and should be considered when performing surgical or endodontic procedures [25]. The implementation of AI-assisted diagnostic tools could enhance preoperative planning, facilitate prompt diagnosis, and mitigate the risk of IAN damage. By providing precise measurements of RA-MC proximity, clinicians can make more informed decisions regarding the necessity and extent of surgical interventions, potentially reducing the incidence of nerve injuries. However, our results show that CephX was not a reliable tool in this particular utilization.

Furthermore, the findings of this study contribute to the growing body of evidence supporting the integration of AI in digital dentistry. As highlighted by Issa et al. [42], the effectiveness of AI in segmentation tasks on CBCT scans is notable. However, our study calls for caution when extending these findings to the assessment of RA-MC proximity. The recent study by Jindanil et al. [30] evaluated the performance of an AI-based tool for mandibular incisive canal segmentation on CBCT scans. The proposed tool enabled an impressive 284-fold time reduction compared to manual segmentation with 85.2% precision, 90.2% recall, 99.8% precision, and 100% consistency. However, the study material consisted of only 20 test images. Further studies are needed to validate these astonishing accuracy metrics. Aside from the issue of CephX’s moderate diagnostic accuracy, our study revealed certain variability in the diagnoses of repeated analyses. This may indicate inconsistency and variability in the performance of the AI platform or the development and improvement of its algorithms during the period between conducting the repeated examination. However, the recent literature shows highly promising results and encourages further research on the development of AI tools. Hopefully, the integration of AI can streamline diagnostic workflows, reduce clinician workload, and enhance the overall accuracy of assessments [43]. This could lead to more predictable and safer dental procedures, ultimately improving patient outcomes.

Our study has several limitations. The relatively small sample size (57 patients aged 18–30 years) of a homogeneous population may limit the generalizability of the findings to the broader patient population undergoing dental procedures. Future research should include a larger and more diverse population, expanding the age range and including patients with varied dental and medical histories to further validate these findings and understand the applicability of the AI tool across different clinical scenarios. Another limitation is that the study material consisted solely of CT images. It is possible that the utilization of CBCT images would yield different results, but to date, no such studies have been published. Additionally, this study did not explore the potential impact of different AI algorithms on diagnostic performance. Different AI models may exhibit varying degrees of accuracy, sensitivity, and specificity, which could influence clinical decision making. Comparative studies assessing multiple AI platforms could provide deeper insights into the strengths and limitations of each tool. Therefore, our results should not be generalized.

Future studies should also explore the integration of AI platforms with other diagnostic modalities, such as MRI, to enhance the comprehensiveness of assessments. Ideally, this would lead to precise, radiation-free diagnostics. Moreover, longitudinal studies are needed to evaluate the long-term clinical outcomes associated with the use of AI-assisted diagnostic tools in dental practice. Understanding how these tools influence patient outcomes over time, including the incidence of complications and patient satisfaction, will be crucial for their widespread adoption.

## 5. Conclusions

This study demonstrated the low diagnostic accuracy of the evaluated AI platform in RA-MC proximity assessment. CephX was unable to provide an accurate diagnosis of RA-MC proximity in our sample of CT examinations.

## Figures and Tables

**Table 1:** Summary of readers’ RA-MC assessments.

Tooth	Missing	Mean	SD	Median	Min	Max	Direct Communication	Frequency [%]
35	1	3.84	1.98	3.54	0.69	9.61	0	0.00
36	6	3.95	2.31	3.61	0.43	12.4	0	0.00
37	3	2.75	2.25	2.24	0	13.4	1	1.85
38	11	1.43	1.56	1.02	0	7.03	11	23.91
45	2	3.68	2	3.44	0	9.62	1	1.82
46	6	3.79	2.56	2.98	0	12.2	1	1.96
47	1	2.3	2.09	1.71	0	12.9	7	12.5
48	12	1.43	1.68	0.95	0	7.68	15	33.33

SD—standard deviation; Q1—lower quartile; Q3—upper quartile.

**Table 2:** Mean RA-MC distances.

Teeth	Mean	SD	Min	Max
Second premolars	3.76	2.81	0.69	9.62
First molars	3.87	3.45	0	12.4
Second molars	2.52	3.07	0	13.4
Third molars	1.43	2.29	0	7.68

SD—standard deviation.

**Table 3:** Diagnostic accuracy metrics of the AI program for RA-MC proximity for three predefined thresholds.

Threshold	Sensitivity	Specificity	Accuracy	PPV	NPV	F1
0 mm	36.36%	86.67%	76.79%	40.00%	84.78%	38.10%
0.5 mm	40.91%	97.06%	75.00%	90.00%	71.74%	56.25%
1 mm	31.25%	100.00%	60.71%	100.00%	52.17%	47.62%

PPV—positive predictive value; NPV—negative predictive value.

**Table 4:** Correlation between mean RA-MC distances and AI’s diagnosis on RA-MC proximity.

AI	N	Distance According to Readers [mm]							*p*
		Mean	SD	Median	Min	Max	Q1	Q3	
Proximity	158	1.96	1.81	1.40	0	6.77	0.50	3.1	*p* < 0.001 *
No proximity	655	3.12	2.31	2.71	0	13.40	1.48	4.3	

*p*—Mann–Whitney test; SD—standard deviation; Q1—lower quartile; Q3—upper quartile. * Statistically significant (*p* < 0.05).

**Table 5:** Interreader concordance for the RA-MC measurements.

Parameter	ICC	95% CI		Agreement (Cicchetti)	Agreement (Koo and Li)
Distance	0.91	0.848	0.968	Excellent	Excellent

ICC—interclass correlation coefficient; CI—confidence intervals.

**Table 6:** Repeatability of AI results for RA-MC proximity diagnoses.

κ	95% CI		Agreement	Interpretation
0.855	0.581	1	94.74%	Strong

κ—Fleiss kappa; CI—confidence intervals.

## Data Availability

Data are available on request.

## References

[1] Zhan C., Huang M., Yang X., Hou J. (2021). Dental Nerves: A Neglected Mediator of Pulpitis. Int. Endod. J..

[2] Dissanayaka W.L., Zhang C. (2017). The Role of Vasculature Engineering in Dental Pulp Regeneration. J. Endod..

[3] Tay A.B.G., Zuniga J.R. (2007). Clinical Characteristics of Trigeminal Nerve Injury Referrals to a University Centre. Int. J. Oral Maxillofac. Surg..

[4] Renton T. (2010). Prevention of Iatrogenic Inferior Alveolar Nerve Injuries in Relation to Dental Procedures. Dent. Update.

[5] Castro R., Guivarc’h M., Foletti J.M., Catherine J.H., Chossegros C., Guyot L. (2018). Endodontic-Related Inferior Alveolar Nerve Injuries: A Review and a Therapeutic Flow Chart. J. Stomatol. Oral Maxillofac. Surg..

[6] McLeod N.M.H., Bowe D.C. (2016). Nerve Injury Associated with Orthognathic Surgery. Part 2: Inferior Alveolar Nerve. Br. J. Oral Maxillofac. Surg..

[7] Sarikov R., Juodzbalys G. (2014). Inferior Alveolar Nerve Injury after Mandibular Third Molar Extraction: A Literature Review. J. Oral Maxillofac. Res..

[8] Alali A.M., Alanzi T.H. (2021). Inferior Alveolar Nerve Damage Secondary to Orthodontic Treatment: A Systematic Scoping Review. Int. J. Risk Saf. Med..

[9] Sayed N., Bakathir A., Pasha M., Al-Sudairy S. (2019). Complications of Third Molar Extraction: A Retrospective Study from a Tertiary Healthcare Centre in Oman. Sultan Qaboos Univ. Med. J..

[10] Lin C.S., Wu S.Y., Huang H.Y., Lai Y.L. (2016). Systematic Review and Meta-Analysis on Incidence of Altered Sensation of Mandibular Implant Surgery. PLoS ONE.

[11] Libersa P., Savignat M., Tonnel A. (2007). Neurosensory Disturbances of the Inferior Alveolar Nerve: A Retrospective Study of Complaints in a 10-Year Period. J. Oral Maxillofac. Surg..

[12] Chong B.S., Quinn A., Pawar R.R., Makdissi J., Sidhu S.K. (2015). The Anatomical Relationship between the Roots of Mandibular Second Molars and the Inferior Alveolar Nerve. Int. Endod. J..

[13] Nguyen E., Grubor D., Chandu A. (2014). Risk Factors for Permanent Injury of Inferior Alveolar and Lingual Nerves during Third Molar Surgery. J. Oral Maxillofac. Surg..

[14] You T.M. (2021). Tooth Hypersensitivity Associated with Paresthesia after Inferior Alveolar Nerve Injury: Case Report and Related Neurophysiology. J. Dent. Anesth. Pain. Med..

[15] Tassoker M. (2019). Diversion of the Mandibular Canal: Is It the Best Predictor of Inferior Alveolar Nerve Damage during Mandibular Third Molar Surgery on Panoramic Radiographs?. Imaging Sci. Dent..

[16] Lübbers H.T., Matthews F., Damerau G., Kruse A.L., Obwegeser J.A., Grätz K.W., Eyrich G.K. (2011). Anatomy of Impacted Lower Third Molars Evaluated by Computerized Tomography: Is There an Indication for 3-Dimensional Imaging?. Oral Surg. Oral Med. Oral Pathol. Oral Radiol. Endodontology.

[17] Castro M.A.A., Lagravere-Vich M.O., Amaral T.M.P., Abreu M.H.G., Mesquita R.A. (2015). Classifications of Mandibular Canal Branching: A Review of Literature. World J. Radiol..

[18] Haas L.F., Dutra K., Porporatti A.L., Mezzomo L.A., De Luca Canto G., Flores-Mir C., Corrêa M. (2015). Anatomical Variations of Mandibular Canal Detected by Panoramic Radiography and CT: A Systematic Review and Meta-Analysis. Dentomaxillofacial Radiol..

[19] Jacobs R., Quirynen M., Bornstein M.M. (2014). Neurovascular Disturbances after Implant Surgery. Periodontology 2000.

[20] MeriÃ P., Naoumova J. (2020). Web-Based Fully Automated Cephalometric Analysis: Comparisons between App-Aided, Computerized, and Manual Tracings. Turk. J. Orthod..

[21] Abesi F., Jamali A.S., Zamani M. (2023). Accuracy of Artificial Intelligence in the Detection and Segmentation of Oral and Maxillofacial Structures Using Cone-Beam Computed Tomography Images: A Systematic Review and Meta-Analysis. Pol. J. Radiol..

[22] Kazimierczak N., Kazimierczak W., Serafin Z., Nowicki P., Jankowski T., Jankowska A., Janiszewska-Olszowska J. (2024). Skeletal Facial Asymmetry: Reliability of Manual and Artificial Intelligence-Driven Analysis. Dentomaxillofacial Radiol..

[23] Puciło M., Lipski M., Sroczyk-Jaszczyńska M., Puciło A., Nowicka A. (2020). The Anatomical Relationship between the Roots of Erupted Permanent Teeth and the Mandibular Canal: A Systematic Review. Surg. Radiol. Anat..

[24] Denio D., Torabinejad M., Bakland L.K. (1992). Anatomical Relationship of the Mandibular Canal to Its Surrounding Structures in Mature Mandibles. J. Endod..

[25] Bürklein S., Grund C., Schäfer E. (2015). Relationship between Root Apices and the Mandibular Canal: A Cone-Beam Computed Tomographic Analysis in a German Population. J. Endod..

[26] Oliveira A.C.S., Candeiro G.T.M., Pacheco da Costa F.F.N., Gazzaneo I.D., Alves F.R.F., Marques F.V. (2019). Distance and Bone Density between the Root Apex and the Mandibular Canal: A Cone-Beam Study of 9202 Roots from a Brazilian Population. J. Endod..

[27] Kovisto T., Ahmad M., Bowles W.R. (2011). Proximity of the Mandibular Canal to the Tooth Apex. J. Endod..

[28] Kawashima Y., Sakai O., Shosho D., Kaneda T., Gohel A. (2016). Proximity of the Mandibular Canal to Teeth and Cortical Bone. J. Endod..

[29] Udupa J.K., McLaughlin D.J., Wu X., Tong Y., Simone C.B., Camaratta J., Torigian D.A., Pednekar G.V., Webster R.J., Fei B. (2018). Image Quality and Segmentation. Proceedings of the Medical Imaging 2018: Image-Guided Procedures, Robotic Interventions, and Modeling.

[30] Jindanil T., Marinho-Vieira L.E., de-Azevedo-Vaz S.L., Jacobs R. (2023). A Unique Artificial Intelligence-Based Tool for Automated CBCT Segmentation of Mandibular Incisive Canal. Dentomaxillofacial Radiol..

[31] Kaasalainen T., Ekholm M., Siiskonen T., Kortesniemi M. (2021). Dental Cone Beam CT: An Updated Review. Phys. Medica.

[32] Fokas G., Vaughn V.M., Scarfe W.C., Bornstein M.M. (2018). Accuracy of Linear Measurements on CBCT Images Related to Presurgical Implant Treatment Planning: A Systematic Review. Clin. Oral Implants Res..

[33] Wikner J., Hanken H., Eulenburg C., Heiland M., Gröbe A., Assaf A.T., Riecke B., Friedrich R.E. (2016). Linear Accuracy and Reliability of Volume Data Sets Acquired by Two CBCT-Devices and an MSCT Using Virtual Models: A Comparative in-Vitro Study. Acta Odontol. Scand..

[34] Kazimierczak N., Kazimierczak W., Serafin Z., Nowicki P., Lemanowicz A., Nadolska K., Janiszewska-Olszowska J. (2023). Correlation Analysis of Nasal Septum Deviation and Results of AI-Driven Automated 3D Cephalometric Analysis. J. Clin. Med..

[35] Alqahtani H. (2020). Evaluation of an Online Website-Based Platform for Cephalometric Analysis. J. Stomatol. Oral Maxillofac. Surg..

[36] Khalid R.F., Azeez S.M. (2022). Comparison of Cephalometric Measurements of On-Screen Images by CephX Software and Hard-Copy Printouts by Conventional Manual Tracing. J. Hunan Univ. Nat. Sci..

[37] Sammartino G., Wang H., Citarella R., Lepore M., Marenzi G. (2013). Analysis of Occlusal Stresses Transmitted to the Inferior Alveolar Nerve by Multiple Threaded Implants. J. Periodontol..

[38] Uğur Aydın Z., Göller Bulut D. (2019). Relationship between the Anatomic Structures and Mandibular Posterior Teeth for Endodontic Surgery in a Turkish Population: A Cone-Beam Computed Tomographic Analysis. Clin. Oral Investig..

[39] Bastien A.V., Adnot J., Moizan H., Calenda, Trost O. (2017). Secondary Surgical Decompression of the Inferior Alveolar Nerve after Overfilling of Endodontic Sealer into the Mandibular Canal: Case Report and Literature Review. J. Stomatol. Oral Maxillofac. Surg..

[40] Jaskari J., Sahlsten J., Järnstedt J., Mehtonen H., Karhu K., Sundqvist O., Hietanen A., Varjonen V., Mattila V., Kaski K. (2020). Deep Learning Method for Mandibular Canal Segmentation in Dental Cone Beam Computed Tomography Volumes. Sci. Rep..

[41] Sert S., Bayirli G.S. (2004). Evaluation of the Root Canal Configurations of the Mandibular and Maxillary Permanent Teeth by Gender in the Turkish Population. J. Endod..

[42] Issa J., Olszewski R., Dyszkiewicz-Konwińska M. (2022). The Effectiveness of Semi-Automated and Fully Automatic Segmentation for Inferior Alveolar Canal Localization on CBCT Scans: A Systematic Review. Int. J. Environ. Res. Public Health.

[43] Waller J., O’connor A., Rafaat E., Amireh A., Dempsey J., Martin C., Umair M. (2022). Applications and Challenges of Artificial Intelligence in Diagnostic and Interventional Radiology. Pol. J. Radiol..

